# Biosafety: From a traditional approach to an integrated approach

**DOI:** 10.3389/fpubh.2022.956623

**Published:** 2022-08-02

**Authors:** Mara Bellati, Vincenzo Russo, Paolo Alberto Leone, Margherita Zito, Aldo Luperini

**Affiliations:** ^1^Institute of Agricultural Biology and Biotechnology, National Research Council (CNR), Milan, Italy; ^2^Department of Business, Law, Economics and Consumer Behaviour “Carlo A. Ricciardi”, IULM University, Milan, Italy; ^3^Behavior and Brain Lab IULM, Center of Research on Neuromarketing, IULM University, Milan, Italy

**Keywords:** biosafety, risk perception, workplace safety, risk assessment, organizational safety

## Introduction

Organizational safety support covering all health and safety policies could provide antidotes to the physical and psychological problem experienced by employees (1). Biosafety is an important issue globally, as a line of defense that protects health personnel, the public and the environment from exposure to hazardous agents. Biosafety refers to the protection, control and accountability measures implemented to prevent the loss, theft, misuse, diversion or intentional release of biological agents, toxins and related resources as well as unauthorized access to, retention or transfer of such material (2). Most developing countries have weak health systems and consequently weak biosafety (3). Even today, there is great uncertainty among practitioners about the correct containment measures when using growth chambers for processed plants. Genetically modified microorganisms (GMMs) are used as vectors for sequences or entire genes, with the aim of silencing endogenous genes, or introducing genes modified to express proteins with characteristics designed by the researcher. Genetic engineering is used to produce vaccines, antibiotics, therapeutic antibodies, resistant or more productive plants or for the development of gene therapies, the treatment of neurological diseases or acquired genetic dysfunctions such as Alzheimer's disease, dystonia, diabetes, multiple sclerosis or arthritis (4–6). The extreme accessibility of GMMs and the latency period (sometimes years) with which some undesirable effects can emerge creates the uncertainty that their use occurs without a thorough awareness of the potential risks associated. In high-risk laboratories unsafe behavior among workers appears to be a critical factor in workplace accidents (7). Unsafe behavior can be motivated by internal and external factors, among which risk perception is a key internal one (8). Research has demonstrated the influence of risk perception on different kinds of safety behavior and involvement in safety management (9). Risk means “uncertainty about and severity of the consequences (or outcomes) of an activity with respect to something that humans value” (10). As risk perception is subjective and depends on a set of values, concerns, or knowledge (11), when workers perceive risk, they are likely to adopt different ways to judge risk. The rational risk perception meaning that workers tend to perceive risk through three rational risk formulations: the probability of risk occurrence, the severity of risk impact, and the expected utility of risk (12). These perceptions or judgment serve as a basis for everyday decision making (13), and are also likely to influence decision making on safety behavior.

## The perception of risk

The perception of risk is personal. In fact, people decide to face or avoid the risk situation in a subjective way (14). Each activity is based on the perception of risk and its more or less conscious evaluation. Moreover, the perceptual process of risk is strongly influenced and conditioned by the emotions generated when discovering and learning about a new danger and what possible harm it can bring. Contrary to what many believe, for humans, risk perception is scarcely dependent on rational factors, such as the use of probability and logic, but on the contrary, it is strongly determined by emotions (15). The personal perception of risk is influenced by habits and previous experience, is based on personal experience or that of others, varies in relation to the collective acceptability of risk which changes over time, places, work groups, cultures and with respect to personal and cultural values, age and gender. It is also influenced by knowledge of hazards, thus the feeling of immunity by those familiar with a given situation, the immediacy of harm, freedom in risk taking, the concentration of harm over time, the harmfulness of the hazards present and their frequency, personal exposure and subjective cost/benefit assessment (12).

Risk propensity increases if events are perceived to be controllable by the subject, so there is a perceived degree of modifiability in actions. Individual type variables such as attitudes toward safety and social type variables such as peer support can influence the likelihood of risk events occurring.

Risk is processed in the mind in two ways:

- Analytical: logical processing of information, based on theoretical knowledge.
- Experiential: automatic, made up of reactions due to the stimulus (through direct or indirect experience) and the emotion it arouses. Experience determines people's ‘perception' of things and the beliefs they hold. These beliefs determine the way they act and the results they achieve.

Psychologists and Sociologists emphasize that risk perception can be irrational and influenced by diverse factors, such as characteristics of risk (16), personal variables (17, 18), as well as cultural and socioeconomic background (19, 20).

## Risk assessment

A Traditional Approach to risk assessment (as shown in Table 1) considers exclusively technical and legislative knowledge to give a definition of risk for each workplace context (21–23). This approach is linked to reference theories to treat risk as a specific factor to be analyzed and managed alone (24) with the main objective to create standardized approaches and models for understanding, assessing, and communicating risk (25). This kind of risk management models and guidelines used exclusively self-report methodologies for analysis and do not take into account soft skills and transversal competences. Traditional risk management models, often are not strongly related with a high level of biosafety, because they do not take into account the organizational context and the decision-making processes of the employee.

**Table 1:** Traditional biosafety risk assessment and integrated biosafety risk assessment.

**Approach**	**Methods**	**Main characteristics**	**Outcome of the approach**
Traditional biosafety risk assessment	Technical and legislative information	Neglect worker and organizational factors	Product guidelines and reference models
Integrated biosafety risk assessment	Technical, legislative and organizational climate information	Add worker and organizational factors	Product guidelines, reference models, tangible best practices in safety climate and safety performance

An Integrated Approach (see Table 1) adds more factors of psychological interest to the traditional studies of risk assessment, which may contribute to correct some errors impacting on risk assessment in a biological laboratory (26). In fact, it takes into account: risk linked personality traits (27); emotional styles (28); empathy and team work capacity (29); cognitive errors and biases (30, 31); cognitive overload and monotonous routine (32); organizational risk communication (33); work-related stress (34); protective and preventive factors (35). They must necessarily be considered as a fundamental part of the risk assessment studies and not set aside as mere secondary variables of risk reference models. All these factors combine to create the need not only to enforce existing regulations and procedures, but also to create best practices to manage the new biosafety challenges in public research and hospitals.

To sum up looking at the table, it is possible see the added value that the Integrated Approach brings to the study of risk assessment. In fact, the Traditional Approach only considers technical and legislative knowledge in the field of biosafety, leaving out the organizational and psychological factors associated with the worker. On the other hand, the added value brought by the use of an Integrated Approach to risk assessment in achieving the outcome is to take into account aspects related to the work organization and the worker himself. Increase the level of safety climate and safety organizational culture could be effective in reducing incidents and improving safety performance indicators (36). The human and organizational factor is essential for the implementation of actions and policies based on the psychophysical wellbeing of the individual and thus on improving performance, organizational wellbeing and safe behavior (37, 38).

## Discussion

The current laboratory safety guidelines published in “Biosafety in Microbiological and Biomedical Laboratories,” 5th ed. (BMBL) (39) for effective biosafety management are derived from significant research has been conducted to understand the physical and psychosocial factors in the workplace that influence behavior, especially job roles, behavioral modeling and feedback, policy enforcement, availability and social support. Once again it is necessary to reiterate the importance of approaching the study of biosafety not only from the traditional - and fundamental - systematic and legislative approach, but it is also essential to investigate those predisposing and preventive factors linked to the cognitive and emotional aspect of workers and work organizations using an Integrated Approach.

Improvements come after significant research conducted to understand the physical and psychosocial factors in the workplace that influence the safe behavior. The effect of general organizational climate on safety performance was mediated by safety climate, while the effect of safety climate on safety performance was partially mediated by safety knowledge and motivation (38).

Risk assessment in biology is a process designed to estimate the risks to human health and the environment to prevent the release of biological agents and toxins. Biotechnology and Biosafety are a heavily discussed issues in almost every country, where opinions of the different parties vary considerably and sometimes are quite different. If you want your organization to change the paradigm of security analysis and prevention, you need to create new experiences and give them new meanings (40). You need to show new ways of working and use new models of thinking to help people develop new tools and keys to safety interpretation (41). To develop motivational and training paths that take into account the perceived risk in a biological laboratory with the aim of making users capable and motivated to manage risk.

Traditional risk assessments should be integrated with organizational and social considerations in order to design and implement risk management strategies able to prevent, reduce or eliminate such risk (42).

## Author contributions

Conceptualization, writing—review and editing, and writing—original draft preparation: MB, MZ, VR, PL, and AL. Supervision: VR, MZ, PL, and AL. Project administration and funding acquisition: VR and AL. All authors have read and agreed to the published version of the manuscript.

## Funding

This study was developed within the project BRIC: Approcci innovativi alla biosicurezza per la tutela della salute dell'uomo e dell'ambiente, funded by INAIL.

## Conflict of interest

The authors declare that the research was conducted in the absence of any commercial or financial relationships that could be construed as a potential conflict of interest.

## Publisher's note

All claims expressed in this article are solely those of the authors and do not necessarily represent those of their affiliated organizations, or those of the publisher, the editors and the reviewers. Any product that may be evaluated in this article, or claim that may be made by its manufacturer, is not guaranteed or endorsed by the publisher.
